# An intractable case of suspected psoriatic arthritis combined with Dupuytren’s disease

**DOI:** 10.12669/pjms.311.6079

**Published:** 2015

**Authors:** Wen Quan Ding, Jian Hui Gu

**Affiliations:** 1 Wen Quan Ding, MD, Department of Hand Surgery, Hand Surgery Research Center, Affiliated Hospital of Nantong University, Nantong, Jiangsu, China.; 2 Jian Hui Gu, MD, Department of Hand Surgery, Hand Surgery Research Center, Affiliated Hospital of Nantong University, Nantong, Jiangsu, China.

**Keywords:** Dupuytren's disease, Psoriatic arthritis, Rheumatoid arthritis

## Abstract

Some cases of psoriatic arthritis (PsA) cannot be explicitly diagnosed, especially when the skin and nail lesions present years after the joint disease or are absent. Autoimmunity may also play a role in the development of Dupuytren's disease. However, the simultaneous presence of PsA and Dupuytren’s disease is very rare. We present a patient displaying arthritis in multiple small joints, with bone erosions and bony fusions in all four extremities, combined with Dupuytren’s disease. Because of the atypical clinical manifestation, the diagnosis perplexed doctors for decades. Without formal treatment, the disease followed a natural course over time. Reviewing the patient’s data, a potential diagnosis of PsA, combined with Dupuytren’s disease, was eventually made. After surgery, contractures of palmar and plantar fascia as well the thumb web were released, and the hallux valgus was corrected.

## INTRODUCTION

Inflammatory arthritis, such as rheumatoid arthritis (RA) and psoriatic arthritis (PsA), usually damages the small joints of the limbs, causing severe disabilities. Although RA and PsA have typical manifestations, confusion may exist between seronegative RA and PsA when the skin and nail lesions present years after the joint disease, or are absent. Approximately 6–18% of PsA cases occur with an average of 7 years before the onset of psoriasis.^1^ With an understanding of how the disease develops, the presence of psoriasis is not mandatory for a PsA diagnosis.^2^ Taniguchi and Kamatani reported a case of PsA without the appearance of psoriatic skin or nail lesions by 21 years.^3^

The occurrence of Dupuytren's disease in the hands of PsA patients is rare. We describe an intractable case that was preliminarily diagnosed with PsA, without typical psoriatic skin lesions, combined with Dupuytren’s disease, including bilateral thumb web and plantar fascia contractures.

## CASE REPORT

A 48-year-old female presented with arthritis in multiple small joints, bone erosions and fusions in both hands and feet, Dupuytren's disease in both hands, contracture of the first web spaces in both hands, contracture of the plantar fascia in both feet, valgus of the first to third metatarsophalangeal joints of her left foot, and ﬂexor tenosynovitis of her left thumb. She had a history of progressive hand and foot deformities that began in 1992 as redness and mild pruritus of her right little finger. At the time, conventional radiography showed arthritis in multiple small joints, with evidence of bone erosions in both hands and feet (Fig. 2 a b). Immunological tests were weakly positive or negative for rheumatoid factor (RF, weakly positive twice in 1993 and negative thereafter). She also tested negative for antistreptolysin O (ASO), and had a normal erythrocyte sedimentation rate (ESR) and normal levels of C-reactive protein (CRP), uric acid (UA), antinuclear antibody (ANA), extractable nuclear antigen (ENA), and immunoglobulin (Ig)G, IgA, and IgM. She was treated with a short course of corticosteroids and immunosuppressive agents, but as her diagnosis was unclear and the treatment was ineffective, she discontinued the therapy. Her only experience with suspicious skin lesions occurred 4 years later in 1996, when skin lesions manifested on the sides of both arms, without the presence of a silver scale. The skin lesion lasted for several months. At the time, her dermatologist did not believe that she met the criteria for a diagnosis of psoriasis. She reported that she had not experienced severe hand pain over the intervening years, which allowed her working in a garment factory. The patient denied a personal or family history of psoriasis or other autoimmune disease.

**Fig.1:**
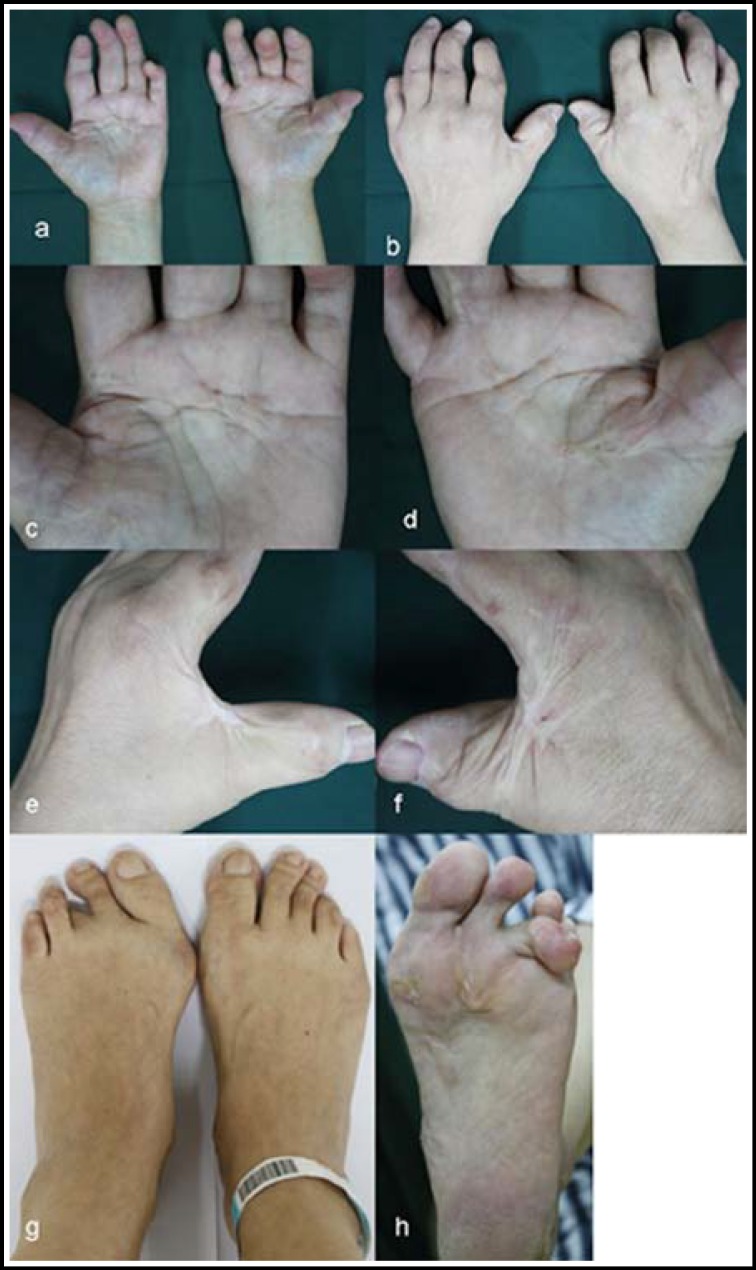
**a and b:** Overview of both hands showing multiple finger malformations in both hands.

A physical examination showed multiple, bilateral deformities of the fingers, bilateral Dupuytren's disease, bilateral contracture of the thumb web and plantar fascia, scar formation on both arms, poor grip and pinch strength in both hands, and limited activity of multiple fingers due to joints stiffness and contractures. The proximal phalanx of her right thumb was short with overlapping skin, and her left thumb snapped when flexed and extended (Fig.1).

**Fig.2:**
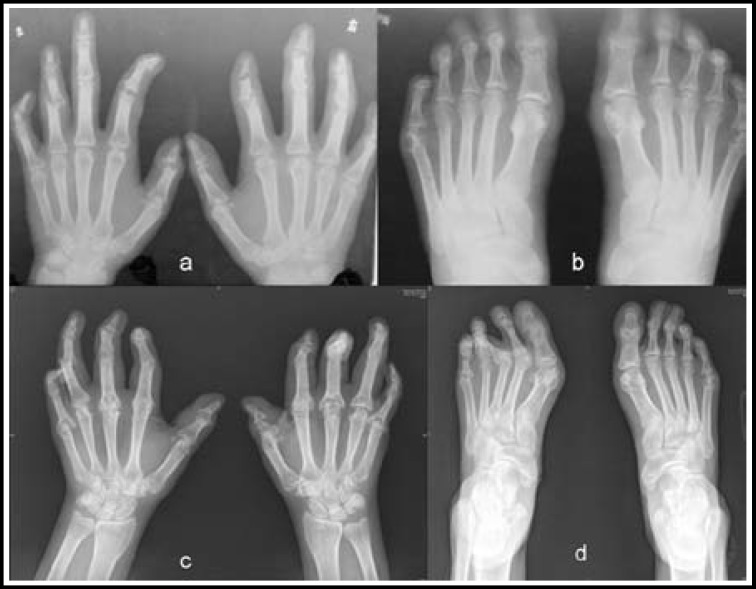
**a and b:** Radiographs obtained in 1992 showed arthritis in multiple small joints, with bone erosion, in both hands and feet.

Radiographs showed evidence of arthritis in multiple small joints. There was also evidence of bone erosion and fusion in both hands and feet, which was limited to the segment from the metacarpal (metatarsal) neck to the tip. Some of these joints also showed subluxation (Fig. 2 c d). Her shoulder, elbow, wrist, hip, knee, ankle, and sacroiliac joints were free of apparent arthritis. Additionally, biochemical and immunological test results were negative for RF and ASO; she also demonstrated a normal ESR and normal levels of CRP, UA, ANA, ENA, human leukocyte antigen (HLA)-B27, anti keratin antibody (AKA), and antibodies directed against cyclic citrullinated peptides (ACCP). A karyotype analysis was performed, showing a normal G-banding analysis in cultured lymphocytes. A potential diagnosis of psoriatic arthritis, combined with Dupuytren’s disease, was made.

The surgical plan involved palmar aponeurotomy; releasing thumb web contractures; partial plantar fascia resection in the left foot; reduction of the left foot first and third metatarsophalangeal joints and fusion with K-wire. After surgery, the patient was followed up for 9 months, and showed release contracture of palmar and plantar fascia as well the thumb web, and the hallux valgus was corrected.

## DISCUSSION

This patient had such a series of complicated disorders that her clinicians were faced with a dilemma as to whether to diagnose seronegative RA or PsA. The American College of Rheumatology/European League Against Rheumatism criteria^4^ are considered positive in patients with the absence of another diagnosis explaining the symptoms, in addition to typical erosions for RA or a score ≥6/10. Our patient scored 6 points, including 5 points for joint involvement (10 joints involved, including at least 1 small joint), and 1 point for synovitis duration (≥6 weeks).

However, a diagnosis of PsA could be more suitable for this patient, even though the patient did not present with typical psoriasis or have a family history of psoriasis. The classiﬁcation criteria created by Fournié et al.^5^ and the Classiﬁcation of Psoriatic Arthritis (CASPAR)^6^ indicated that psoriasis was not mandatory for PsA diagnosis, which were reported had high sensitivity and speciﬁcity. According to the classiﬁcation criteria for PsA made by Fournié, with a diagnostic score cut-off of 11 points, this patient received 12 points for demonstrating arthritis of a distal interphalangeal joint (3 points), characteristic radiographic criteria (5 points), and being RF-negative (4 points). The criteria of Classiﬁcation of Psoriatic Arthritis (CASPAR) is inﬂammatory articular disease (joint, spine, or entheseal) with additional 3 or more manifestations. This patient marched that: inﬂammatory articular disease of joints; a negative test for rheumatoid factor; history of dactylitis; radiological evidence of juxta-articular new bone formation. Hence, this patient met the criteria for a PsA diagnosis, regardless of whether her skin lesions were diagnosed as psoriasis. Thus, a potential diagnosis of psoriatic arthritis, combined with Dupuytren’s disease, was given.

Dupuytren’s disease is characterized by the development of new fibrotic tissue in the form of nodules and cords. Evidence for a role of the HLA system in Dupuytren’s disease suggests that the immune system plays a role in the disease.^7^ Enthesitis is common in PsA patients. In the present patient, enthesitis was an important pathology that explained the ‘pencil-in-cup’ deformity in multiple metacarpophalangeal and interphalangeal joints due to bone erosion and bony fusion^2^, and her ﬂexor tenosynovitis and toes valgus. Queiro et al.^8^ suggested that HLA-DR17 is associated with enthesitis in PsA. Thus, the autoimmune mechanisms involving the HLA system may provide connections between PsA and Dupuytren’s disease.

By the time of the patient’s first clinical visit in 1992, the degree of destruction of her small joints was already serious, but she discontinued therapy. Fortunately, the disease only damaged some of the small joints in her hands and feet, sparing the large joints, allowing her to continue working and performing the activities of daily living. The natural disease process, observed in this patient, suggests that the disease may be self-limiting.

We can learn something from this interesting case. First, in addition to immunologic testing and radiography, PsA can be diagnosed early using magnetic resonance imaging or ultrasonography, before the joints are damaged. If patients are diagnosed and treated by modern techniques early, formation of destructive changes of joints can be delayed or even avoided.^9^ Second, surgery for saving hands function should be performed as soon as possible when joints damage is stabilized. If tendons were not affected, arthroplasty may be a reasonable choice. Third, although the disease may be self-limiting, the patient could get a better outcome if she continued regular internal medicine therapy from 1992, when her metacarpophalangeal joints were not involved (Fig.2 a).

**c and d:** Bilateral Dupuytren's disease. Clinical manifestations in the right hand are more obvious.

**e and f:** Bilateral contracture of the first web space. The proximal phalanx of the right thumb is short and has overlapping skin.

**g and h:** Toes valgus and contracture of the plantar fascia of the left foot.

**c and d:** Radiographs obtained in 2013 showed arthritis in multiple small joints, with bone erosions and fusions, in both hands and feet. The arthritis was limited to the segment from the metacarpal (metatarsal) neck to the tip. Some of these joints indicated subluxation.

In summary, we report an intractable case of suspected PsA, without typical psoriatic skin or nail lesions, combined with Dupuytren’s disease, which was preliminarily diagnosed by classiﬁcation criteria in the absence of the classic psoriatic skin lesions. The autoimmunity mechanisms associated with both diseases may provide connections between PsA and Dupuytren’s disease. An early diagnosis may be helpful for preventing joint deterioration.
